# Case Report: Venous pulsatile tinnitus induced by enlarged oblique occipital sinus and resultant diverticulum/dehiscence of the sigmoid-jugular wall

**DOI:** 10.3389/fsurg.2022.1014649

**Published:** 2023-01-06

**Authors:** Yue-Lin Hsieh, Shenjiang Wang, Wuqing Wang

**Affiliations:** ^1^ Department of Otology and Skull Base Surgery, Eye Ear Nose & Throat Hospital, Fudan University, Shanghai, China; ^2^ Key Laboratory of Hearing Science, Ministry of Health, Shanghai, China; ^3^ Department of Radiology, Eye Ear Nose & Throat Hospital, Fudan University, Shanghai, China

**Keywords:** pulsatile tinnitus, occipital sinus, oblique occipital sinus, diverticulum, dehiscence

## Abstract

Pulsatile tinnitus (PT) caused by enlarged oblique occipital sinus (OOS) and resultant diverticulum/dehiscence of the sigmoid-jugular wall has not been described in previous literature. This study recruits one case of PT induced by ipsilateral enlarged OOS and sigmoid-jugular wall diverticulum (case 1) alongside one case of PT induced by ipsilateral enlarged OOS and sigmoid-jugular wall dehiscence (case 2). Various radiologic and computational techniques including computed tomography (CT), magnetic resonance (MR) imaging, Doppler ultrasound, and computational fluid dynamics (CFD) simulation were implemented. Transmastoid sinus wall reconstruction was performed on case 1 with a large sigmoid-jugular diverticulum potentially traumatizing the facial nerve canal. Contrast-enhanced CT or MR venogram images coupling with three-dimensional reconstructed are advantageous in revealing the covert route of OOS that runs under the cerebellum and drains directly into jugular bulb (JB) region. PT in case 1 was successfully eliminated after transmastoid sinus wall reconstruction surgery. Tinnitus handicap inventory score in case 1 reduced from 70 to 0. The ipsilateral jugular outflow mean velocity (V_mn_) and flow volume (F_VOL_) were 42.5 cm/s and 25.9 g/s (case 1 prior to surgery) and 56.6 cm/s and 41.2 g/s (case 2), respectively. Based on CFD simulation, the peak flow velocity in OOS was 1.85 m/s and 2.1 m/s, the wall pressure of the diverticular dome and dehiscence area of the SS-JB wall was 1724.7 Pa and 369.8 Pa in case 1 and 2, respectively. Enlarged OOS caries greater flow kinetic energy that possibly induces sigmoid-jugular wall diverticulum/dehiscence; transmastoid surgical method is safe and therapeutically effective against PT induced by enlarged OOS.

## Introduction

Vascular pulsatile tinnitus (PT) or pulse-synchronous tinnitus is the auditory perception of vascular sounds in line with the cardiac rhythm (1). The most common type of vascular PT is venous PT (2), which is characterized by the reduction or elimination of PT sound when the ipsilateral internal jugular vein (IJV) is compressed (3).

Venous PT requires prudent examination of venous vasculature and the surrounding osseous structure near the hearing apparatus. The causes of venous PT include sigmoid sinus (SS) wall anomalies, high-riding jugular bulb (JB) dehiscence/diverticulum, transverse-SS enlargement, and mastoid emissary vein (4, 5). The symptomology and radiologic signs in patients with venous PT overlap with those in patients with idiopathic intracranial hypertension, a clinical entity characterized by increased intracranial pressure with unknown cause (4). Hypothesized mechanisms of the cause of PT relate to (1) osseous defects surrounding the pulsating vessel that produce vibroacoustic noises, and (2) increased flow kinetic energy that engenders hydroacoustic vascular sounds (6). The produced mechanical sound is transmitted predominantly through the air-conduction pathway to the inner ear, resulting in the perception of PT (5–7).

The occipital sinus is a small venous channel found in 64.5%–95% of cadaveric specimens and normal subjects (8). The length and inner diameter of an occipital sinus measure between 15 and 70 mm and 0.33–7.06 mm, respectively, and age-related regression may occur (9). It is usually positioned underneath the torcular herophili at the midline near the attachment of the falx cerebelli or deviates on the left or right side with two or more cranial or caudal divisions. In a large case series, Kobayashi et al. found that 11.9% of the occipital sinus communicates with or branches into the SS (10). This type of occipital sinus is called oblique occipital sinus (OOS). However, despite previous reports, the epidemiology of a large OOS that runs directly into the SS or the JB remains unknown.

This study reports unprecedented causes of venous PT caused by ipsilateral OOS and sigmoid-jugular wall anomalies (SJWAs) invading the mastoid cavity and sheds light on the mechanism of this newly discovered phenotype of venous PT.

## Case presentation

### Case report 1

**Figure 1:**
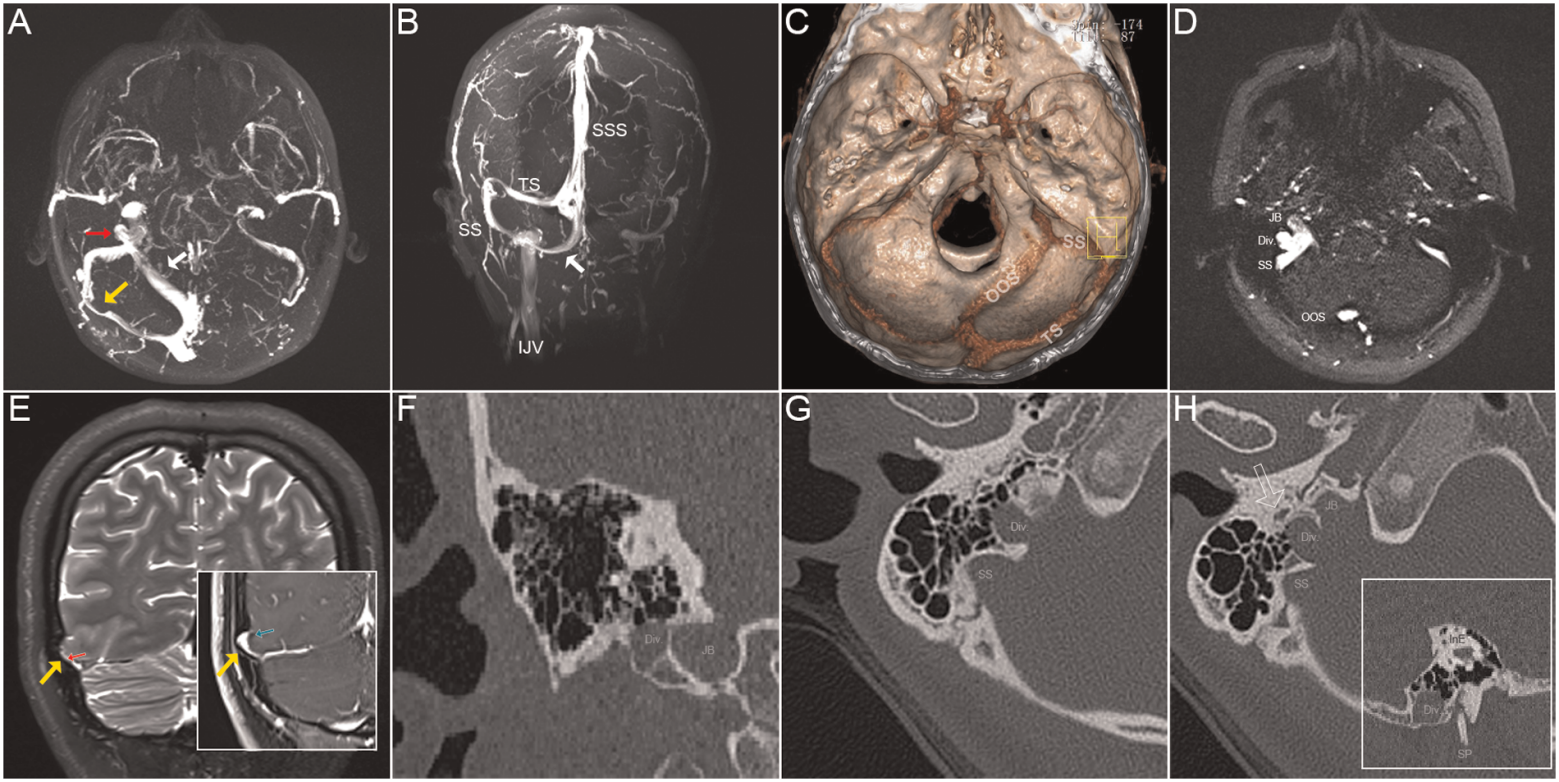
Radiological imaging and signs of case 1 of right-sided pulsatile tinnitus. (**A**) The maximum intensity projection (MIP) reconstruction of axial 2D-time-of-flight (TOF) magnetic resonance (MR) images showing an enlarged oblique occipital sinus (OOS, white arrow) anastomosing with the lower curve sigmoid sinus (SS), forming a diverticulum (red arrow) in between the SS and jugular bulb (JB). Note the abrupt transverse sinus stenosis at the middle segment. (**B**) Coronal MIP reconstruction of 2D-TOF MR images. The white arrow indicates the enlarged OOS. (**C**) Volume rendering technique reconstructed using postoperative contrast-enhanced CT images demonstrating an enlarged OOS running above the occipital bone and underneath the cerebellum to directly anastomose with SS. (**D**) An axial 2D-TOF MR slice showing the SS, diverticulum (Div.), JB, and OOS. (**E**) Coronal T2-weighted MR and T1-weighted 2D FLASH images showing the brain herniation into arachnoid granulation [(cerebrospinal fluid signal hyperintense on T2 (red arrow) and hypointense on T1 (green arrow)] that compress into the transverse sinus lumen (yellow arrow), which should be suspected of increased intracranial pressure. (**F**) Coronal CT image showing the relative location of the diverticulum and JB. (**G**) Axial CT image exhibiting a seemingly right-sided high-riding JB dehiscence/diverticulum (pseudo-JB anomalies sign) invading the mastoid cavity. The bulging vascular wall emerging at the lower SS was a diverticulum (Div.) but not JB. (**H**) Axial CT slice showing the SS, diverticulum (Div.), and JB. The hollow arrow indicates the facial nerve canal. The reconstructed sagittal CT slice shows the diverticulum (Div.) nearly exited the mastoid apex. SP and InE indicate the styloid process and inner ear, respectively.

**Figure 2:**
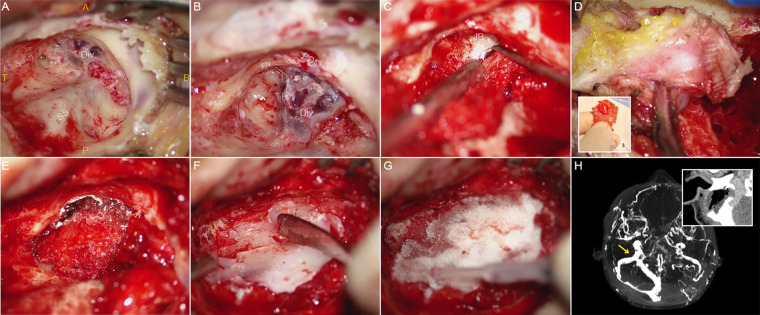
Intraoperative photographs and detailed description of the transmastoid surgery of case 1. (**A**) Localizing the SS-JB diverticulum by adopting a lower mastoid bone incision site with the upper and lower bonds leveling with the base of the external canal and the mastoid apex, respectively. Note the absence of osseous wall and dura mater covering the surface of the diverticulum upon initial exposure. SS, sigmoid sinus; Div., diverticulum; A, anterior; *P*, posterior; T, top; B, bottom. (**B**) Skeletonization of the posterior semi-circular canal (PSC), facial nerve (FN), and the diverticulum (Div.) in sequential order. Notably, the diverticulum abuts the posterior digastric muscle (PDM) and nearly exits the mastoid apex. (**C**) A 45-degree knife curette was used to separate the diverticular wall from the surrounding osseous walls. To prevent excessive hemorrhage, a ribbon gauze was placed topically over the diverticulum to protect its fragile surface. (**D**) Rupture of the diverticular wall can be inevitable during the separation procedure, which the high blood flow velocity and pressure render the process of hemostasis arduous. Therefore, we harvested and customized the hemostatic material (a robust yet pliable artificial dura mater-temporalis fascia complex) that also serves as the sinus wall repairing material. (1) Topical fixation of a tough solidified artificial dura mater smeared with surgical glue to compress and reduce the diverticulum. (2) Robust yet pliable hardened/dried temporalis fascia providing a petticoat-like supporting structure to fit and laminate with the surrounding osseous structures. (3) Prior to holistic and surgical glue fixation of the repairing materials, the suture was sewn at the caudal portion of the temporalis fascia to prevent suction of the operative filler into the vascular laceration and into the sigmoid sinus. (**E**) Surgical glue fixation of the repairing materials covering the diverticulum. The thickness of repairing materials should be prudently controlled to avoid total collapse of the sigmoid sinus wall. (**F**) and (**G**) Bone wax-solidified gelatin sponge complex is applied topically to reconstruct the sound-proof barrier. (**H**) Postoperative contrast-enhanced CT and magnetic-resonance venogram demonstrating complete reduction of the diverticulum. The yellow arrow exhibiting the befitted extraluminal compression over the sigmoid sinus.

A 43-year-old female patient with persistent right sided PT for 1 year as the solitary symptom presented to our facility in June 2022. Her PT was abolished when the ipsilateral IJV was compressed. Endoscopic examination revealed a normal tympanic membrane in both ears. Pure-tone audiometry showed no hearing loss on either side. Computed tomography (CT) revealed a right-sided SS-jugular diverticulum invading the lower mastoid cavity, potentially traumatizing the facial nerve and nearly exiting the mastoid apex (Figure 1). The closest distance between the dome of the diverticular and facial nerve canal was 3 mm. Magnetic resonance (MR) images revealed an enlarged OOS running into the lower curve of the right-side SS, with ipsilateral intrinsic transverse sinus stenosis and contralateral transverse sinus hypoplasia. A diverticulum had formed between the lower curve of the SS and JB. Fundus examination showed presence of bilateral papilledema. The tinnitus handicap score was 70. Computational fluid dynamics (CFD) was performed to analyze sinus flow hemodynamics (see Supplementary Digital Content S1 for CFD methodology). Transmastoid surgical resurfacing of SJWAs was performed to resolve the PT (see Figure 2 for a detailed description of the surgical procedures).

### Case report 2

**Figure 3:**
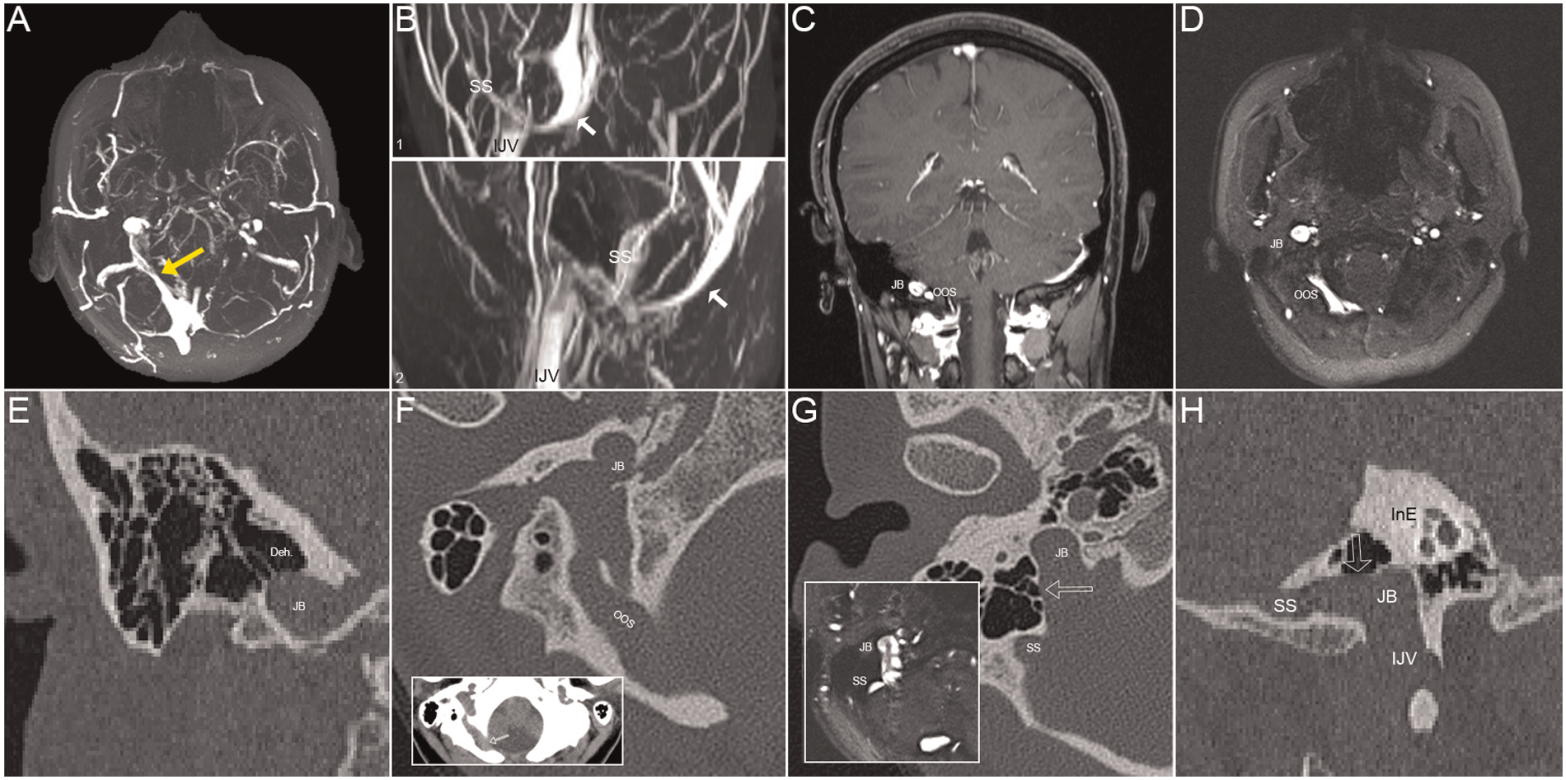
Radiological images of the patient in case 2 of right-sided pulsatile tinnitus (PT). (**A**) The maximum intensity projection (MIP) reconstruction of axial 2D-time-of-flight (TOF) magnetic resonance (MR) images showing an enlarged oblique occipital sinus (OOS) with distal stenosis (yellow arrow) anastomosing with the ipsilateral jugular bulb (JB). (**B**) (1) Coronal and (2) sagittal maximum intensity projection (MIP) reconstruction of 2D-time-of-flight (TOF) magnetic resonance (MR) images demonstrating the enlarged OOS (white arrows) running under the cerebellum. SS, sigmoid sinus; IJV, internal jugular vein. (**C**) Coronal contrast-enhanced T1-weighted gradient echo (2D FLASH) MR slice showing the relative locations of OOS and JB. (**D**) An axial 2D-TOF MR slice showing the SS, diverticulum (Div.), JB, and OOS. (**E**) Coronal CT image demonstrating dehiscence at the top of the SS-JB wall. (**F**) The notch of the OOS cuts through the edge of the foramen magnum of the occipital bone (red arrow). (**G**) Axial CT image showing SS-JB wall dehiscence and intrasinus flow pattern exhibited using the corresponding 2D-time-of-flight (TOF) magnetic resonance slice. (**H**) Reconstructed sagittal CT image exhibiting SS-JB wall dehiscence and locations of the SS, JB, and inner ear (InE).

A 35-year-old female patient complained of persistent right-sided PT for a duration of 1.5 years visited our clinic in October 2019. Her PT was silenced during ipsilateral IJV compression. Pure-tone audiometry revealed no hearing loss in either ear. Endoscopic examination revealed insignificant findings on both sides. CT images showed discontinuity and absence of bone structure in the SS-JB wall (Figure 3). MR resonance imaging revealed an enlarged OOS anastomosed with the JB and bilateral transverse sinus hypoplasia. Fundus examination results were normal. The tinnitus handicap index score was 22. CFD simulation was performed. Since the patient chose not to undergo surgical treatment, an annual radiologic follow-up was advised, and continuous online medical consultation was provided.

## Results

**Figure 4:**
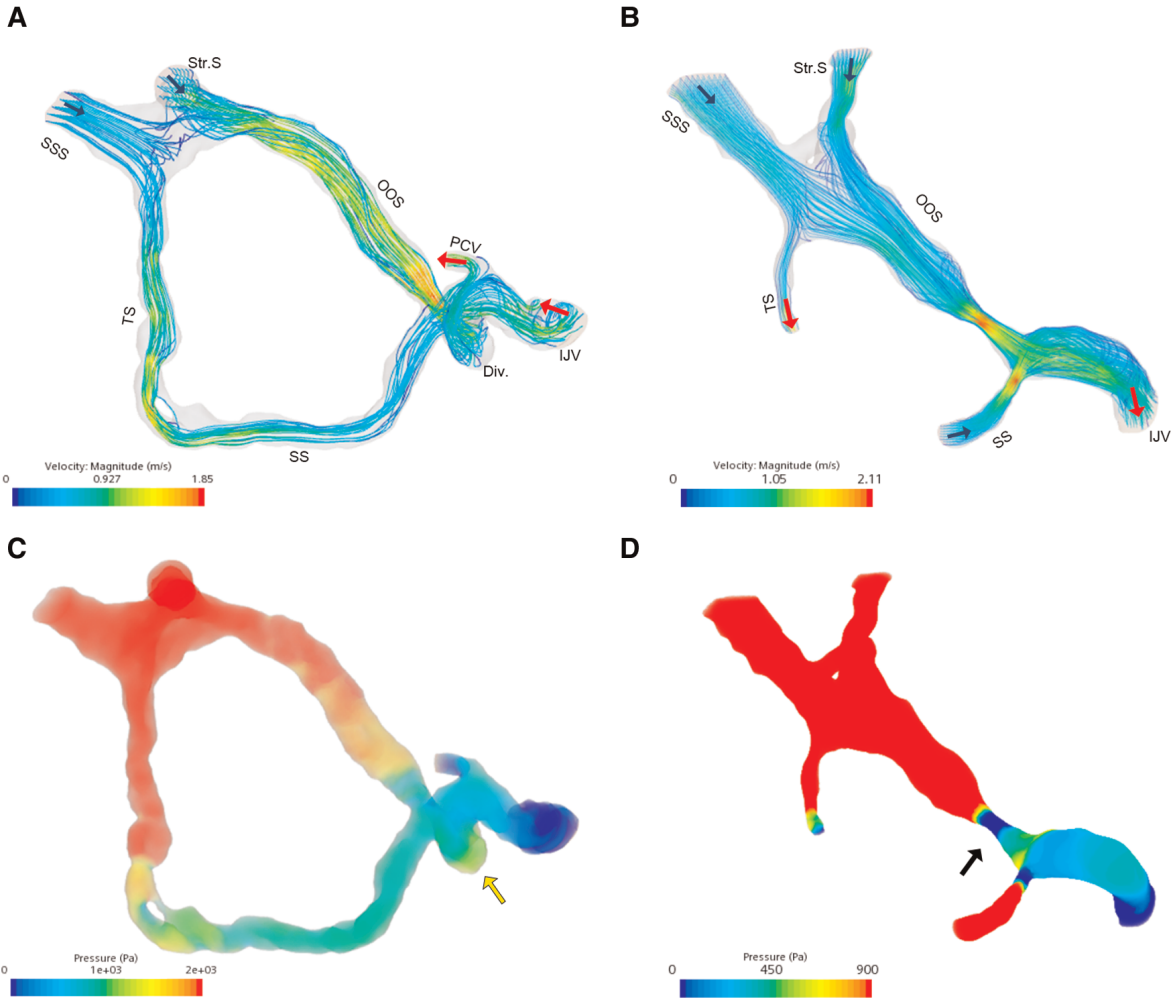
Flow field analysis of the enlarged oblique occipital sinus. (**A**) Velocity flow field in case 1. (**B**) Velocity flow field in case 2. (**C**) Flow pressure field of case 1. Note the pressure increase at the diverticular dome (yellow arrow). (**D**) Flow pressure field of case 2. Note the large trans-stenotic pressure gradient caused by OOS stenosis. Blue and red arrows indicate flow inlet and outlet, respectively. SSS, superior sagittal sinus; Str.S, straight sinus; TS, transverse sinus; OOS, oblique occipital sinus; SS, sigmoid sinus; Div., diverticulum; PCV, posterior condylar vein; IJV, internal jugular vein.

The ipsilateral jugular outflow mean velocity (V_mn_) and flow volume (F_VOL_) were 42.5 cm/s and 25.9 g/s (case 1) and 56.6 cm/s and 41.2 g/s (case 2), respectively. The postoperative ipsilateral V_mn_ and F_VOL_ of case 1 were 44.1 cm/s and 26.9 g/s. CFD analysis found that the peak velocity in the enlarged OOS was 1.85 m/s and 2.1 m/s, the wall pressure of the diverticular dome and SS-JB wall was 1724.7 Pa and 369.8 Pa in case 1 and 2, respectively (Figure 4).

PT was eliminated after surgery in case 1. The cross-sectional diameter of the diverticular and SS lumen was reduced from 1.49 to 5.4 mm, rendering a smooth SS silhouette. The postoperative tinnitus handicap index score was 0. The patient had ear numbness and a plugged ear after surgery. There was no recurrence of PT or symptoms related to increased intracranial pressure during the 6-week follow-up.

## Discussion

To the best of our knowledge, PT related to SJWAs caused by an enlarged OOS has not been described in the literature. An enlarged OOS can be overlooked owing to its covert route underneath the cerebellum and direct drainage into the SS or JB. In this study, OOS dominated over the ipsilateral or bilateral hypoplastic transverse sinus and carried excessive flow kinetic energy that exerted flow pressure onto the lower curve of the SS wall. The onset of PT in these two cases likely resulted from the formation of SJWAs, since the presence of OOS is congenital (8, 9). This inference is akin to the mechanism of PT caused by SS wall anomalies, which frequently occur on the dominant side of the transverse-sigmoid sinus junction. Additionally, the Venturi effect was also evidenced by the middle to distal stenosis of the OOS, producing a jet flow effect similar to that observed in transverse sinus stenosis (5). A more severe degree of osseous wall erosion and vascular wall protrusion was observed in these two cases, which is conceivably related to the high flow kinetic energy carried by the enlarged OOS.

According to the jugular Doppler measurement, the ipsilateral F_VOL_ of subjects with enlarged OOS was 1.5∼2.5 times larger than those with venous PT caused by SS wall anomalies (7). Additionally, CFD indicates that the vortex generated at the intersection of the OOS and SS increased the regional flow pressure gradient. Given the impaired flow condition, patients with an enlarged OOS should be suspected of having increased intracranial pressure, especially when bilateral hypoplastic transverse sinus is present. Therefore, surgical attempts to occlude the OOS lumen or significantly reduce the venous pool volume may be unwise, whereas the stenting of OOS stenosis has been shown therapeutically effective, although the presence of SJWAs was not evaluated in that study (11).

PT secondary to SJWAs induced by an enlarged OOS can be treated with transtemporal sinus wall reconstruction surgery. Unlike transverse-SS junction diverticulum or dehiscence, SJWAs caused by an enlarged OOS can be more strenuous to operate on because of its location adjacent to vital anatomical structures. Owing to the fragile diverticulum surface, separating the diverticular and osseous walls (typically the medial portion) most likely lacerates the vascular surface and causes considerable hemorrhage. Because SJWAs are located at the lower curve of the SS, we tailored the repair material and inserted it into the epidural space to stanch bleeding during the reduction of the diverticulum. To avoid sinus overcompression or the entrance of hemostat debris into SS, haemostatic materials should be customized with certain malleability, robustness, and retractability; in our case, this was a large piece of temporalis muscle with the solidified artificial dura mater fixated on the muscular edge sewn with suture. Lastly, regarding strong OOS flow impaction, we reduced the diverticulum and applied multilayer materials to prevent further invasion of the diverticular wall into other anatomical structures and preclude the hydroacoustic/vibroacoustic sound transmission in the mastoid cavity.

## Conclusion

Enlarged OOS caries greater flow kinetic energy (with 1.5∼2.5 times larger F_VOL_ compared to those with venous PT without the afferent enlarged OOS) that possibly induces SJWAs. Although the presence of enlarged OOS is rare, this study reveals that the transmastoid surgical method safe and therapeutically effective against PT induced by enlarged OOS and resultant diverticulum/dehiscence of the sigmoid-jugular wall.

## Data Availability

The original contributions presented in the study are included in the article/**Supplementary Material**, further inquiries can be directed to the corresponding author/s.
