# Supplementary material for: Case Report: Venous pulsatile tinnitus induced by enlarged oblique occipital sinus and resultant diverticulum/dehiscence of the sigmoid-jugular wall
Source: Front Surg. 2023 Jan 6;9:1014649. doi: 10.3389/fsurg.2022.1014649 (PMC9852330; doi:10.3389/fsurg.2022.1014649)
Supplement: Supplementary file 1 [file Datasheet1.pdf]

Supplemental Digital Content 1

Computational fluid dynamics analysis was performed for case 1 and 2. The 3D computational vascular models were reconstructed using patient-specific magnetic resonance venogram images, Mimics 19.0 and 3-Matic 11.0 software (Materialise, Belgium). Sinus branches < 5 mm were removed. A total number of 623682 and 644,832 cells were established for flow simulation of case 1 and 2, respectively. The boundary layer thickness was 0.4 mm, and 5 layers were created.

For the flow simulation, the continuity equation and Navier–Stokes equations were solved using the stable laminar method using software Star-CCM+ 2020 (Siemens, Germany):

$$\nabla \cdot \mathbf{u} = 0$$

$$\rho \frac{\partial \mathbf{u}}{\partial t} + \rho \mathbf{u} \cdot \nabla \mathbf{u} = -\nabla p + \mu \nabla^2 \mathbf{u},$$

where the dynamic viscosity μ is 0.00345 Pa s, the blood density ρ is 1,050 kg/m^3, and **u** is the velocity vector of the incompressible Newtonian blood flow. Zero pressure was set for all vascular outlets. Individual velocity inlets were established based on the mean flow velocity measured at the upper internal jugular vein by the Doppler ultrasound MyLab Class C (ESOATE SpA, Genoa, Italy) with an LA-523 transducer (4–13 MHz).
